# Stay home, save lives: Characterizing sickness presenteeism and motives among healthcare personnel in the COVID-19 pandemic

**DOI:** 10.1017/ash.2022.137

**Published:** 2022-05-16

**Authors:** Amanda Brown Marusiak, David Weber, Cynthia Culbreth, Erica Pettigrew, Lisa Stancill, Emily Sickbert-Bennett

## Abstract

**Background:** Working while ill, or presenteeism, has been documented at substantial levels among healthcare personnel (HCP) along with its consequences for both patient and HCP safety. Limited literature has been published on HCP presenteeism during the COVID-19 pandemic, and specific motivations for this behavior are not well described. Understanding both individual and systemic factors that contribute to presenteeism is key to reducing respiratory illness transmission in the healthcare setting. We characterized the frequency of and motivations for presenteeism in the workforce of a large academic medical center during the COVID-19 pandemic. **Method:** We deployed a voluntary, anonymous electronic survey to HCP at University of North Carolina (UNC) Medical Center in December 2021, which was approved by the UNC Institutional Review Board. We received 591 responses recruited through employee newsletters. Respondents recounted their frequency of presenteeism since March 2020, defined as coming to work feeling feverish plus cough and/or sore throat. In total, 24.6% reported presenteeism at least once, with 8.1% reporting twice and 5.3% 3 or more times. Asking more generally about any symptoms while working, the following were most common: headache (26%), sinus congestion (20%), sore throat (13%), cough (13%), and muscle aches (9.3%). **Results:** Motivations for presenteeism fell broadly into 4 categories: (1) perception of low risk for COVID-19 infection, (2) concerns about workplace culture and operations, (3) issues with sick leave, and (4) concerns about employment record and status. Among HCP reporting at least 1 instance, the most common motivations for presenteeism included feeling low risk for COVID-19 infection due to mild symptoms (59.9%), being vaccinated (50.6%), avoiding increasing colleagues’ workload (48.3%), avoiding employment record impact (39.6%), and saving sick days for other purposes (37.9%). Asked to identify a primary motivation, 40.3% reported feeling low risk for COVID-19 infection due to mild symptoms or vaccination, 21.2% reported a workplace culture issue (ie, increasing colleague workload, perception of weakness, responsibility for patients), 20.6% reported sick leave availability and use (including difficulty finding coverage) and 17.8% reported employment record ramifications including termination. **Conclusions:** This survey coincided with 2the onset of the SARS-CoV-2 ο (omicron) variant locally, and as such, risk perceptions and motivations for presenteeism may have changed. Responses were self-reported and generalizability is limited. Still, these results highlight the importance of risk messaging and demonstrate the many factors to be considered as potential presenteeism motivators. Mitigating these drivers is particularly critical during high-risk times such as pandemics or seasonal peaks of respiratory illness.

**Funding:** None

**Disclosures:** None

